# Left Common Iliac Vein Stenting in a Case of Postural Orthostatic Tachycardia Syndrome/Pelvic Pain Overlap

**DOI:** 10.7759/cureus.53974

**Published:** 2024-02-10

**Authors:** Mary M Pelling, Matthew T Brown, Charles A Gilliland, Alexis Cutchins

**Affiliations:** 1 Internal Medicine, Emory University School of Medicine, Atlanta, USA; 2 Cardiology, Emory University School of Medicine, Atlanta, USA; 3 Interventional Radiology, Piedmont Henry Hospital, Stockbridge, USA; 4 Cardiology, Emory University Hospital Midtown, Atlanta, USA

**Keywords:** may-thurner syndrome, pelvic pain, venous disease, iliac vein stent, pelvic congestion syndrome, postural orthostatic tachycardia syndrome (pots)

## Abstract

Postural orthostatic tachycardia syndrome (POTS) is mainly characterized by orthostatic intolerance and positional tachycardia although it frequently involves a myriad of non-specific symptoms that seem to overlap with existing medical conditions. Recent efforts have been made to further classify subtypes of POTS and associated conditions to better delineate underlying pathophysiology in an effort to guide diagnosis and tailor treatment. Here, we present a 22-year-old female with debilitating symptoms of POTS who reported pelvic pain on review of systems and underwent vascular ultrasound of the inferior vena cava, iliac veins, and bilateral lower extremities which revealed the characteristic left common iliac vein compression of May-Thurner syndrome prompting venous stenting which provided systemic symptomatic relief.

## Introduction

Postural orthostatic tachycardia syndrome (POTS) is a heterogeneous disorder with a substantial impact on quality of life. Clinically, this condition is defined as orthostatic intolerance (OI) associated with a heart rate increase of 30 beats per minute (bpm) or a rate that exceeds 120 bpm within the first 10 minutes of standing [1]. Treatment regimens are extremely patient-specific and primarily focus on symptom improvement and gradual exercise tolerance. Recently, there has been an investigation into the overlap between venous disease and orthostatic intolerance with 69% of patients with POTS found to have significant (>50%) left common iliac vein compression compared to 40% of age-matched healthy controls [2]. Iliac vein compression, also known as May-Thurner syndrome (MTS), occurs when the left common iliac vein is compressed between the right common iliac artery and lumbar spine. This syndrome is twice as common in women than in men and is present in about 1/3 of the general population [3,4]. More recently, pelvic venous disease, which includes iliac vein compression, has been associated with chronic pelvic pain, including chronic low back pain. Importantly, 63% of patients with diagnosed pelvic venous disease when surveyed reported symptoms of dizziness and OI compared to 1% in the general population [5].

Pelvic venous disease (PVD) is thought to be the cause of at least 30% of patients presenting with chronic pelvic pain and iliac vein compression and/or gonadal (ovarian) vein reflux have been found to be the most common underlying anomalies [4]. The etiology of PVD related to iliac vein compression involves impeded venous outflow due to iliac vein compression by the right iliac artery, resulting in elevated sacral venous plexus, ascending lumbar, and epidural venous pressure, as well as cross-pelvic collaterals. This results in compromised venous return, pooling of deoxygenated blood, and possibly irritation of neurovascular bundles leading to symptoms of chronic pelvic pain, which can be successfully treated with iliac vein stenting [6-8]. Here, we present a case of a young female meeting the objective and clinical criteria for POTS with OI found to have iliac vein compression on vascular ultrasonography for pelvic pain whose systemic symptoms greatly improved after iliac vein stenting.

## Case presentation

A 22-year-old female with a past medical history of attention deficit and hyperactivity disorder (ADHD), polycystic ovarian syndrome (PCOS), and Tourette syndrome was referred to cardiology for evaluation of POTS. On presentation, her chief complaint was positional pre-syncope with tunnel vision and loss of hearing upon standing as well as pain in her lower extremities. Her review of systems was positive for dysphagia, lactose intolerance, seasonal allergies, occasional urticaria, daytime sleepiness, pelvic pain, and joint hypermobility. She was prescribed guanfacine for her ADHD, norethindrone and spironolactone for her PCOS, and clonazepam for her Tourette syndrome. On examination, her blood pressure was 107/65 and her supine heart rate was 63 bpm with an increase to 101 bpm upon standing, associated with lightheadedness and tunnel vision. Laboratory evaluation was unremarkable.

**Figure 1:**
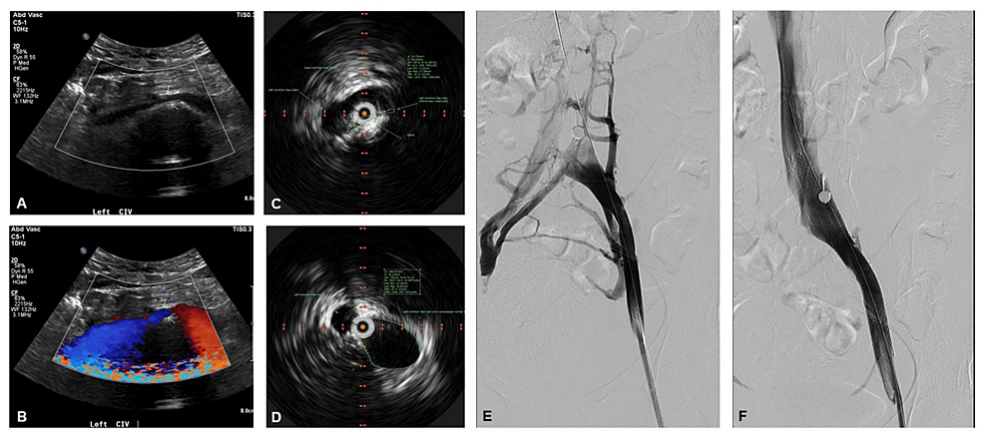
Relief of a May-Thurner Like Left Common Iliac Vein Compression by Venous Stenting. (A) 2D vascular ultrasound illustrating the left common iliac vein compression (arrows) by the right iliac artery (above left) and lumbar spine (below) with (B) turbulent flow in the area by color Doppler assessment. (C) Baseline IVUS demonstrating a minimum lumen area of 12 mm^2^ at the area of venous compression outlined in green compared to (D) 158 mm^2^ beyond the area of compression on pullback. (E) Baseline pelvic venogram revealing obstruction to antegrade flow and presence of collaterals. (F) Post-stent venogram demonstrating restoration of antegrade flow up the inferior vena cava and minimal collateral reflux. IVUS: intravascular ultrasound.

With her description of pelvic pain and postural bilateral lower extremity pain, vascular ultrasonography of her inferior vena cava, iliac veins, and bilateral lower extremities was pursued and revealed compression of the left common iliac vein between the right common iliac artery and lumbar spine (Figure 1A, 1B) without any evidence of deep venous thrombosis. Based on this positive imaging finding and her pelvic pain, she underwent bilateral pelvic venography with intravascular ultrasound (IVUS) which further characterized a severe obstruction of the left common iliac vein with near-complete flow arrest and robust filling of cross-pelvic and para-spinal collateral veins (Figure 1C-1E, Video 1). Intervention was pursued via femoral vein access with initial pre-dilation of the left common iliac vein with a 12 mm x 40 mm conquest balloon before delivering a 12 mm x 140 mm Cook Zilver stent across the compression. Final venogram plus IVUS assessment confirmed marked improvement in flow through the stent with decreased reflux into collateral vessels (Figure 1F, Video 1).

**Video 1:** An Annotated Compilation of Baseline and Post-Stenting Pelvic Venography and Intravascular Ultrasound.

She tolerated the procedure well and at a six-week follow-up reported significant improvement in not only pelvic pain but also orthostatic intolerance and frequency of tics allowing for participation in athletics such as pickleball and de-escalation of psychiatric therapy. These improvements were reflected in her supine-to-standing heart rate increase from 50 to 75 bpm, no longer meeting the criteria for POTS, with resolution of OI symptoms.

## Discussion

This young female’s case of underlying venous compression manifesting as POTS and pelvic pain emphasizes the need for further characterization of POTS symptoms and subtypes to better dictate diagnostic assessment and ideal treatment design for this population that is largely overlooked. Santoshi et al. (2018) found that 80% of patients with PVD had iliac vein compression [6]. It was recommended this outflow be treated with iliac stent placement first prior to embolization of pelvic varices and/or gonadal vein embolization for chronic pelvic pain. If patients were still symptomatic after iliac stent placement and had gonadal vein reflux, embolization was recommended as patients were found to have symptomatic improvement. Iliac vein stenting has been shown to be safe and effective in post-thrombotic and left lower extremity edema as well [9].​​

In a recent case series reported, three patients with Ehlers-Danlos syndrome (EDS) who met diagnostic criteria for POTS were also experiencing symptoms of PVD prompting pelvic imaging revealing severe compression of the left common iliac vein by the right common iliac artery consistent with MTS [10]. The first patient underwent stenting of the left common iliac vein, the second underwent iliac vein stenting as well as gonadal vein embolization, and the third was only beginning to contemplate intervention. Each patient who underwent intervention experienced significant improvement in OI symptoms as well as quality of life much in the same way our patient benefited shortly after her procedure.

Therapies vary widely for POTS, and successful treatment may be viewed as an art focused on symptom improvement and treatment of comorbid or underlying conditions rather than a standardized algorithm [11]. Associations with multiple disorders have been identified, such as EDS described above and more recently COVID, and may provide insight into previously unrecognized etiologies, novel pathophysiology, and optimal management of patient symptoms [10-13].

## Conclusions

POTS is characterized by OI; however, the disorder is frequently accompanied by a constellation of non-specific symptoms that may overlap with other existing medical conditions. As therapies for POTS vary widely and focus primarily on patient-specific symptom relief, it is important to recognize key symptoms that may allow for POTS subtyping and/or recognition of associated conditions to guide diagnostics and tailor treatment. In this case, the key overlap of pelvic pain in POTS raised suspicion for an associated pelvic venous condition and prompted ultrasonography of pelvic vasculature revealing the left common iliac vein compression consistent with MTS for which venous stenting led to significant relief of both OI and pelvic pain.
